# Medicine Availability and Prescribing Policy for Non-Communicable Diseases in the Western Balkan Countries

**DOI:** 10.3389/fpubh.2017.00295

**Published:** 2017-11-08

**Authors:** Tanja Pekez-Pavlisko, Maja Racic, Srebrenka Kusmuk

**Affiliations:** ^1^Family Medicine Clinic “Tanja Pekez-Pavlisko”, Kutina, Croatia; ^2^Faculty of Medicine, Department for Primary Health Care and Public Health, University of East Sarajevo, East Sarajevo, Bosnia and Herzegovina

**Keywords:** family medicine, Western Balkan, chronic non-communicable diseases, prescribing policy, prescribingrelated competencies

## Abstract

**Background:**

During the transition processes, the Western Balkan countries were affected by conflicts and transition-related changes. Life expectancy in these countries is lower, while the mortality from non-communicable diseases (NCDs) is higher in comparison with western and northern parts of Europe. The primary aim of this study was to analyze the treatment possibilities for the most common NCDs in the Western Balkan countries. The secondary aim was to understand and compare the policies regarding prescribing-related competencies of family physicians.

**Methods:**

In June and July 2017, a document analysis was performed of national positive medicines lists, strategic documents, and clinical guidelines for the treatment of the most frequent NCDs; arterial hypertension, diabetes, hyperlipidemia, asthma, and chronic obstructive pulmonary disease (COPD). All text phrases that referred to medicines prescribing were extracted and sorted into following domains: medicine availability, prescribing policy, and medication prescribing-related competencies.

**Results:**

Possibilities for treatment of arterial hypertension, diabetes, hyperlipidemia, asthma, and COPD vary across the Western Balkan countries. This variance is reflected in the number of registered medicines, number of parallels, and number of different combinations, as well as restrictions placed on family physicians in prescribing insulin, inhaled corticosteroids, statins and angiotensin II receptor blockers (ARBs), without consultant’s recommendation.

**Conclusion:**

Western Balkan countries are capable of providing essential medicines for the treatment of NCDs, with full or partial reimbursement. There are some exceptions, related to statins, newer generation of oral antidiabetic agents and some of the antihypertensive combinations. Prescribing-related competences of family physicians are limited. However, this practice is not compliant to the practices of family medicine, its principles and primary care structures, and may potentially result in increased health-care financial ramifications to both the system and patients due to frequent referrals to the specialists.

## Introduction

After the adoption of the Declaration in Alma Ata in 1978, great efforts have been made worldwide regarding the improvement of living conditions (water, electricity, roadways, and other infrastructure), development of primary health care, and vaccination of children (1). All the aforementioned has led to a decline in mortality rate in countries of all levels of development as well as increased life expectancy. Longer lifespan, urbanization, and lifestyle changes result in an increase in morbidity and mortality from non-communicable diseases (NCDs).

According to World Health Organization (WHO) data, of the 57 million global deaths in 2008, 36 million (63%) were due to NCDs, mainly cardiovascular diseases, diabetes, cancer, and chronic respiratory diseases. As the impact of NCDs increases, and as population’s age, annual NCD deaths are projected to continue to rise worldwide, and the greatest increase is expected to be seen in low- and middle-income regions (2).

While popular belief presumes that NCDs afflict mostly high-income populations, evidence shows a very different story. Nearly 80% of NCD deaths occur in low-and middle-income countries and NCDs are the most common causes of death in most countries, except in Africa. With this in mind, First Global Ministerial Conference on Healthy Lifestyles and Non-communicable Diseases Control in Moscow (2011) resulted in Moscow Declaration Preamble (3), followed by a session of the United Nations General Assembly (4), which adopted a number of conclusions of vital importance for primary health care such as to include prevention and control of NCDs among priorities in national health strategies and plans; to revitalize primary health care and promote access to cost-effective interventions for NCDs, including access to essential medicines and technologies and to mobilize additional resources and support innovative approaches to financing essential NCDs health-care interventions within primary health care.

For many decades, Eastern and Southern Europe have had lower life expectancy than the rest of Europe. This was particularly noticeable during the transition processes; however, the countries of the Western Balkan were affected not only by transition related changes but also by conflicts. Hence, besides poverty, transition and conflict have also weakened the health indicators of the Western Balkan countries (5–8). Such conditions were sustained by inadequate or practically non-existent health care reform which did not adapt to the new trends of globalization. A major problem for all newly established countries is decision-making within the health sector that is not based on evidence.

Prior to the disintegration of Yugoslavia, primary health care was at a very high level owing to the work of Andrija Štampar and Ante Vuletić. The latter, at the beginning of the 1960s, introduced the specialty of family medicine which served as a model for the UK, Canada, and other countries. Unfortunately, since 1995, in the Western Balkan countries, family medicine has not had satisfactory position (9–11).

Considering that, along with NCD prevention, both diagnostics and treatment are of utmost importance, the role of regulatory agencies and insurance funds in health policy became vital. Each of the Western Balkan countries has its particular list of medications prescribed by the national insurance fund. Furthermore, each country has regulatory agencies for placing and controlling prescribed medications. Given that some of the countries are small and do not have sufficient capacities, problem with medication control arises after they are placed on the market, especially in Bosnia and Herzegovina and Montenegro (12).

Last but not least, as early as Barbara Starfield’s work was brought to light, it was evident that a well-organized primary health care resulted in better health indicators and lower expenditure (13, 14). In countries that have allowed the progress of family medicine and competences of family physicians, there is a decrease in referral to secondary health care and more comprehensive health care. This is particularly important in the treatment of NCDs since the teamwork of a family physician and other health-care professionals is not only favorable for the treatment of patients but also for primary and secondary prevention of both NCDs and infectious diseases, as well as of consequences caused by violence and accidents.

The underlying principle of well-performing primary health care system is to ensure access to essential medicines for treatment of NCDs; however, availability of medicines is not sufficient to provide continuous care required for patients. Very little is know about prescribing policy for NCDs in Western Balkan countries, which share legacy of the former Yugoslavia in management and financing patterns of health care system. Through document analysis, we aimed to analyze the treatment possibilities for the most common NCDs in countries of the Western Balkan. The questions that guided our research were: to what extent essential medicines from WHO list are included into positive medicines lists of these countries and what is the policy regarding prescribing-related competencies of family physicians.

## Materials and Methods

### Setting

The qualitative exploratory study on prescribing policy was conducted by analyzing documents of Health Insurance Funds from Bosnia and Herzegovina (BiH), Croatia, Macedonia, Montenegro, Serbia, and Slovenia. The basic functions of the Health Insurance Funds are to manage the system finances (compulsory health insurance is the main source health care) and provide legal and managerial support to insure with regard to health and health care. According to the legislative requirements, fund develops and maintains database related to health-care activity and insurance coverage. Insurance coverage includes public or private sectors employees, the retired people, the disabled, and the students, while stateless persons and social care recipients are subsidized by the state budget for the uninsured. All patients have the same rights, regardless of the insurance payment level required. Within the financing of health care, the medications listed in positive medicines list are included. Medications appearing on the list are divided into several separate categories, with specific coverage rate, such as reduced, normal, or preferential reimbursement rate provided for each category. The revision is carried out every few years or more frequently, depending on health expenditure level or public needs. The lists are seen as national documents; therefore, we included them into document analysis to gain a deeper understanding of the prescribing policy and develop empirical knowledge (15).

### Design

To cover the knowledge utilization of the documents, six criteria were formulated (16), while four-step process was performed for conceptualizing the document analysis (17). Additions to a knowledge base were the information derived from Model List of Essential Medicines, provided by the World Health Organization (WHO) (18) for all countries. The essential medicines were defined as medicines with safety, effectiveness, availability, and rational use (19). The focuses of research were three domains: medicine availability, prescribing policy, and medication prescribing-related competencies of family physicians regarding treatment of most common NCDs. Medicine availability included essential medicines for the treatment of the most common NCDs: arterial hypertension, hyperlipidemia, diabetes, asthma, and chronic obstructive pulmonary disease (COPD) as well as management of pain at the end of life. Second domain involved analysis of legislative criteria and policy tools that have been used in controlling pharmaceutical spending. Medication prescribing competency framework is defined as a collection of competencies central to effective, rational, and safe prescribing, based on the judgment and ability to make decision rationally for the benefit of patients (20). The analysis covered angiotensin-converting enzyme (ACE) inhibitors, beta blockers, ARBs, oral hypolipidemic agents, oral hypoglycemic agents, Insulin, opiods, and proton pump inhibitors (PPIs).

### Procedures

A set of positive medicines lists and strategic documents were retrieved through internet searches in June and July 2017. Due to the political divisions in BiH into two entities (The Republic of Srpska and Federation of Bosnia and Herzegovina) and canton levels (10 cantons in Federation, each with different legislation), the research included lists of the Republic of Srpska (RS) and two Federal cantons, Sarajevo, and Herzegovina Neretva. In the RS, authority over health care system is centralized with administration, financing and decision-making policy held by Ministry of Health and Social Welfare, while in Federation of BiH health care system administration is decentralized with each of 10 cantonal ministries having responsibilities for provision and financing of health care at all levels (Federal Ministry of health has limited role that ensures compliance with entity policy regulations). We also retrieved national clinical guidelines for the treatment of the chronic diseases in research focus and clinical practice guidelines of official professional associations (e.g., European Society of Cardiology). All documents were made available at the research sites. A document browser was used to interactively specify queries on the data. To prove the documents’ authenticity, the content of each document has been examined.

### Analysis

Credibility, accuracy, and representativeness of the selected information were determined. The first author (Tanja Pekez-Pavlisko) skimmed (superficially examined) and then systematically red retrieved documents. All text phrases that referred to medicines prescribing were extracted and sorted into following domains: medicine availability, prescribing policy, and medication prescribing-related competencies.

The meaningful and relevant data were identified during first-pass review and separated from the non pertinent text. Selected data were re-reviewed and themes construction was performed. Parallel, co-authors (Maja Racic and Srebrenka Kusmuk) individually analyzed documents. The results were compared and the doubts concerning the inclusion or position of data were discussed. The final results represent consensus between all researchers.

## Results

Medicines availability varied widely, while the prescribing policy and prescribing policy tools often were not corroborated by scientific approach and national as well as international guidelines.

Possibilities of treatment of arterial hypertension vary across the Western Balkan countries. This variance is reflected in the number of registered medication, number of parallels, and number of different combinations, as well as restrictions placed on family physicians in prescribing certain medication without referral to a clinical specialist. Table 1 demonstrates the number of categorized medication on insurance lists per country.

**Table 1:** Number of medicines for treatment of hypertension according to groups, countries, and cantons.

	Bosnia and Hercegovina (Herzegovina Neretva Canton)	Bosnia and Hercegovina (The Republic of Srpska)	Bosnia and Hercegovina (Sarajevo Canton)	Montenegro	Croatia	Former Republic of Yugoslavia Macedonia	Slovenia	Serbia
Diuretics	5	6	5	2	5	4	5	7
Beta blockers	1	4	6	3	6	4	7	6
Angiotensin-converting enzyme (ACE) inhibitors	7	8	6	7	8	2	8	10
Angiotensin II receptor blockers (ARB) inhibitors	1	2	1	5	6	1	5	3
Ca channel blockers	3	6	3	3	8	3	7	6
Combination ACE inhibitors + diuretics	4	6	4	7	7	0	6	7
Combinations ARB inhibitors + diuretics	0	1	2	1	5	1	3	2
Combinations ACE inhibitors + Ca channel blockers	0	1	1	0	3	0	2	2

Croatian and Slovenian medication lists contain several additional combinations, ACE inhibitors, diuretics, calcium channel blockers, beta blockers, and statins, the display of which would decrease the transparency of basic therapeutic groups of medicine for treatment of hypertension.

Furosemide and hydrochlorothiazide are on the positive medicine list in all countries, whereas spirinolactone is not on the list only in Montenegro. Regarding diuretics, there are no restrictions set on their prescriptions for family physicians, except for torasemide, restricted in the Sarajevo and Herzegovina Neretva cantons. In Sarajevo canton, this medication can be prescribed by certified family physicians, while in Herzegovina Neretva Canton, recommendation of a clinical consultant is requested.

**Figure 1:**
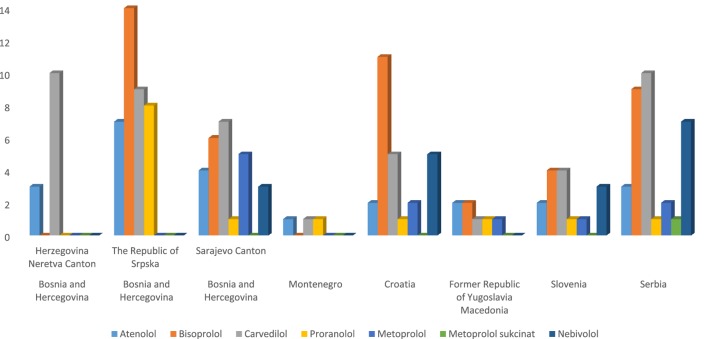
Number of parallels of individual beta blocker in countries and cantons.

The following beta blockers: atenolol, bisoprolol, propranolol, metoprolol, metoprolol succinate, and nebivolol were found to be on positive lists of all countries (Figure 1). Prescribing-related restrictions for beta blockers in family practice are presented in Table 2.

**Table 2:** Prescribing-related restrictions for beta blockers in family practice.

Medicine	Restriction in country/canton	
Atenolol	No restrictions	

Bisoprolol	Sarajevo Canton	Medication can be prescribed by family physicians, specialists of occupational medicine, pediatricians, gynecologist, pulmonologist, internists, and emergence medicine specialists

	Former Republic of Yugoslavia Macedonia	Chronic heart failure, arterial hypertension, and angina pectoris

Carvedilol	Sarajevo Canton	Only for heart failure

	Former Republic of Yugoslavia Macedonia	Chronic heart failure, arterial hypertension, and angina pectoris—on the recommendation of a cardiologist or internist

	Serbia	For heart failure treatment, it is necessary to consult cardiologist, for arterial hypertension treatment not

Proranolol	No restrictions	

Metoprolol	No restrictions	

Metoprolol succinate	Serbia	Chronic heart failure, hypertension, and angina pectoris treatment; it is necessary to consult cardiologist

Nebivolol	Sarajevo Canton	Indications: 1. chronic heart failure, internist’s recommendation is requested; 2. hypertension and angina pectoris

Situation with multiple registered products of various companies is similar in the area of ACE inhibitors and ARB as well as their combination with diuretics. In Table 3, only medicines with most parallels are shown. Lisinopril and hydrochlorothiazide are charged additionally in the RS, as well as other combinations with hydrochlorothiazide (ramipril, irbesartan). ARB inhibitors and its combinations are additionally charged in Serbia and in Croatia for several brand medicines only. ACE inhibitors do not meet criteria for prescribing restrictions in family practice, while there are several restrictions for ARBs (Table 4).

**Table 3:** Number of ACE and ARB inhibitor parallels; combination of ACE with diuretics, combination of ARB with diuretics.

	Bosnia and Hercegovina (Herzegovina Neretva Canton)	Bosnia and Hercegovina (The Republic of Srpska)	Bosnia and Hercegovina (Sarajevo Canton)	Montenegro	Croatia	Former Republic of Yugoslavia Macedonia	Slovenia	Serbia
Enalapril	10	11	5	1	1	4		7
Lisinopril	9	10	6	1	11	1	4	5
Perindopril	0	0	0	0	3	0	11	5
Ramipril	0	16	6	1	10	0	5	7
Losartan	0	0	0	1	7	2	6	7
Valsartan	0	0	5	0	8	0	6	3
Enalapril + hydrochlorothiazide	9	9	5	1	1	0	3	4
Lisinopril + hydrochlorothiazide	8	11	6	1	10	0	4	3
Ramipril + hydrochlorothiazide	0	9	4	1	7	0	4	6
Losartan + hydrochlorothiazide	0	3	0	1	5	0	7	3
Valsartan + hydrochlorothiazide	0	4	0	0	5	0	8	5

*ACE, angiotensin-converting enzyme (ACE) inhibitors, ARB, angiotensin II receptor blockers*.

**Table 4:** Prescribing-related restrictions for angiotensin II receptor blockers in family practice.

Medicine	Restriction in country/canton	
Combination with diuretics	Serbia	Indicated if target values are not achieved *via* monotherapy after 3 months

Losartan	Croatia	For patients intolerant to angiotensin-converting enzyme (ACE) inhibitors and having a cough at least 4 months

	Serbia	For treatment of arterial hypertension and for patients whose ejection fraction is <40%Cardiologist’s or internist’s recommendation requested

Valsartan	Herzegovina Neretva Canton	For patients intolerant to ACE inhibitors, per internist’s recommendation

	Croatia	For patients intolerant to ACE inhibitors and after cough lasting 4 months

Serbia	For treatment of arterial hypertension, for patients whose ejection fraction is <40%, cardiologist’s or internist’s recommendation requested

Ibersartan	The Republic of Srpska	For patients with side effects of ACE inhibitors, per consultant’s recommendation

Croatia	For patients intolerant to ACE inhibitors and having a cough for at least 4 months

Oral hypolipidemic agents have different prescription mechanism in different countries and cantons. Table 5 shows how many parallels an individual statin have and Table 6 regulations regarding their prescribing.

**Table 5:** Number of parallels of oral hypolipidemic agents per country.

Medicine	Bosnia and Hercegovina (Herzegovina Neretva Canton)	Bosnia and Hercegovina (The Republic of Srpska)	Bosnia and Hercegovina (Sarajevo Canton)	Montenegro	Croatia	Former Republic of Yugoslavia Macedonia	Slovenia	Serbia
Simvastatin	0	8	6	1	11	0	7	8
Atorovostatin	0	17	7	1	11	1	14	7
Fluvastatin	0	0	1	0	3	0	2	0
Pravastatin	0	0	0	0	0	0	2	2
Rosuvastatin	0	6	3	0	7	0	10	9
Nicotinic acid	0	0	0	0	0	1	0	0
Ciprofibrate	0	0	0	1	0	0	0	1
Ezetimibe	0	0	0	0	1	0	6 (ezetimib alone or in combination with statin)	1
Fenofibrate	0	0	0	0	5	0	2	0
Cholestyramine	0	0	0	0	1	0	0	0

**Table 6:** Prescribing-related restrictions for oral hypolipemic agents in family practice.

Medication	Restrictions in country/canton	
Statins	Serbia	(a) Medication completely free of charge for patients with inheritable hyperlipidemia, per recommendation by Clinic for endocrine diseases, diabetes, and metabolism disease Clinical center of Serbia (b) Patient partially charged for medication in case of previous myocardial infarction or stroke and as prevention of further occurrence

	The Republic of Srpska	(a) Secondary prevention of coronary disease (b) Diabetes mellitus with hyperlipidemia (c) Chronic kidney failure and condition of transplanted organ with hyperlipidemia

	Sarajevo Canton	In primary prevention for patients who after 3 months of non-pharmacological treatment still has a value of total cholesterol above 7 mmol/L

	Former Republic of Yugoslavia Macedonia	Patients with high cardiovascular risk and LDL cholesterol greater than 3.5 mmol/L (a) Verified coronary arterial disease (myocardial infarction, stabile angina, bypass). Cardiologist’s or internist’s recommendation requested (b) Verified diabetes, family physician prescribe independently (c) Stroke, per neurologist’s and internist’s recommendation (d) Verified coronary artery disease, stenosis >60%, per neurologist’s and internist’s recommendation (e) Patient with 10-year cardiovascular risk >20% according to Framingham score, or >5% according to SCORE model, family physicians are allowed to prescribe without consultant’s recommendation

	Croatia	For secondary prevention in patients with myocardial infarction, ischemic cerebrovascular insult, transitory ischemic attack, carotid occlusive disease and peripheral artery disease, and coronary disease For patients with total cholesterol value greater than 7 mmol/L after three months of non-pharmacological treatment

Statins		For secondary prevention of cardiovascular diseases in patients with total value of total cholesterol >4.5 mmol/L and LDL >2.5 mmol/L. For primary prevention when total cardiovascular risk >20%, if total cholesterol value is >5 mmol/L and LDL cholesterol >3.0 mmol/L For patients with familial hypercholesterolemia

	Montenegro	For patients with myocardial infarction and cerebrovascular insult

Fibrates	Montenegro	Clinical consultant’s recommendation requested

	Croatia	Prescribed only if, after 3 months of non-pharmacological treatment, triglycerides in blood are no less than 2 mmol/L

	Serbia	For patients with familial hypercholesterolemia Clinical consultant’s recommendation requested

Ezetimibe	Croatia	For treatment of primary hypercholesterolemia in patients with very high or high cardiovascular risk who have, despite statin therapy, LDL cholesterol levels ≥ 2.5 mmol/L Clinical consultant’s recommendation requested

Number of parallels of oral antidiabetic agents in countries and cantons is presented in Table 7. The majority of oral antidiabetic agents are prescribed with no restrictions for family practice, except for DPP-4 inhibitors and long-Acting Glucagon-Like Peptide 1 Receptor Agonists (GLP1 agonists). GLP1 are on the lists of The RS, Sarajevo Canton, Croatia and Slovenia.

**Table 7:** Number of parallels of oral antidiabetic agents in countries and cantons.

	Bosnia and Hercegovina (Herzegovina Neretva Canton)	Bosnia and Hercegovina (The Republic of Srpska)	Bosnia and Hercegovina (Sarajevo Canton)	Montenegro	Croatia	Former Republic of Yugoslavia Macedonia	Slovenia	Serbia
Metformine	8	11	4	1	6	1	4	6
Glibenclamid	3	4	2	0	1	0	0	1
Glimepiride	8	10	6	1	7	1	1	3
Repaglinide	0	0	2*	0	5*	1	2	1 E
Gliclazide	0	3	0	1	6	0	3	5
Pioglitazone	0	0	0	0	2**	0	1	1 EK
Gliquidone	0	0	0	0	1	0	1	0
DPP4 inhibitors and SGLT2 inhibitors	0	6 EK1	0	0	13^#^	0	16^##^	0

Treatment of diabetes mellitus type 2 (DM2) faces a large variance and financial capabilities across countries/cantons. Insulin, according to the positive medicine lists, can be prescribed by family physicians only in Croatia, Slovenia, and RS (Table 8).

**Table 8:** Prescribing-related restrictions for insulin in family practice.

Country	Insulin type	Restrictions
Bosnia and Hercegovina (Herzegovina Neretva Canton)	Human insulin	Clinical consultant’s recommendation requested

	Lispro, Aspart, and Glulisine	Diabetologist’s recommendation requested

	Glargine and Detemir	Clinical consultant’s recommendation requested and under special prescription regime

Bosnia and Hercegovina (The Republic of Srpska)	All insulins	No restrictions, however, patient is obligated to keep a journal for administrating insulin (journal can be acquired from the Fund)

Bosnia and Hercegovina (Sarajevo Canton)	Human, Lispro, Aspart, and Glulisine	Clinical consultant’s recommendation requested

	Glargine and Detemir	Clinical consultant’s recommendation requested

		For patients with unregulated glycemia (and HbA1C <6.5%), using oral antidiabetic agents

Montenegro	All insulins	Clinical consultant’s recommendation requested

Croatia	Aspart	Without consultant’s recommendation and within a guideline
		For patients with diabetes on intensive insulin therapy and unregulated glycemia

	Human insulin	Without consultant’s recommendation and without guidelines

	Glulisine	Without consultant’s recommendation and within a guideline
		For patients with diabetes on intensive insulin therapy and unregulated glycemia

	Lispro	Without consultant’s recommendation and with a guideline: for patients with diabetes on intensive insulin therapy and unregulated glycemia

	Glargin	Without consultant’s recommendation and with a guideline: for patients in intensive insulin therapy (1 or 2 daily injections of basal insulin + 3 injections of shortly-acting insulin alongside main meals), who during the past 6 months, despite changes in therapy scheme, fail to achieve satisfactory glicoregulation (HbA1c <6.5%), who have more than one hypoglicemia episode weekly, and who fail to achieve glycemia control with other types of insulin

	Detemir	Without consultant’s recommendation and within a guideline
		For patients on intensive insulin therapy (1 or 2 daily injections of basal insulin+3 injections of shortly acting insulin alongside main meals), who during the past 6 months, despite changes in therapy scheme, fail to achieve satisfactory glucoregulation (HbA1c <6.5%), who have more than one hypoglycemia episode weekly, and who fail to achieve glycemia control with other types of insulin

Former Republic of Yugoslavia Macedonia	Insulin and analogs	Per consultant’s recommendation under the Macedonian Government program

Slovenia	Detemir, Glargine, and Degludek	Only for patients with other hypoglycemic and other insulin

Serbia	Aspart, Glargine, Detemir, and Lispro	Hypoglycemia must be confirmed in a health-care institution (the remainder of restriction explanation is too great for to be included)

	Human	Per internist’s, pediatrician’s or endocrinologist’s recommendation

Possibilities of treating asthma and COPDs are also defined by guidelines and different fund restrictions. For example, salbutamol, aminophyline, and theophylline can be independently prescribed by family physicians in all doses. Salbutamol, as well as theophylline, is available with the exception of RS and Macedonia. The RS included aminophylline in their positive medicines list. Sarajevo Canton, Herzegovina Neretva Canton, Montenegro, and Macedonia do not reimburse for long-acting beta 2 agonists. Inhaled corticosteroids, as well as its combinations with long-acting beta agonists (multiple brands) are available in all countries, but can be prescribed independently by family physicians only in Croatia and Slovenia. Al l countries have ipratropium bromide and tiotropium bromide on their lists. In Serbia, only combination of fenoterol and ipratropium bromide can be prescribed by family physicians, while the treatment with other inhaled medicines (except for salbutamol) needs to be recommended by consultants (e.g., patients in Macedonia need to be referred to asthma or COPD center). Montelukast is not available in the RS and Macedonia. There are no restrictions toward prescribing of this drug in family medicine of Croatia and Slovenia; however, only in Slovenia it can be prescribed as monotherapy, while in other countries it is indicated as additional therapy. Newer medications for treatment of asthma and COPD are available only in Slovenia and Croatia.

The indications for PPIs differ between the countries. In Slovenia and Croatia, duration of therapy is not limited and consultant’s recommendation is not required. In Croatia, there are guidelines for prescribing, but gastroprotection as an indication is not included. PPIs in Montenegro, Serbia, and parts of BiH can be prescribed only for duodenal or gastric ulcer treatment, while in Macedonia, Health Insurance Fund also reimburses treatment of gastro esophageal reflux if diagnosed with endoscopy. There are many parallels of PPIs in all countries (e.g., 17 paralels of pantoprazole in the RS, 13 in the Herzegovina canton, 16 in Croatia).

Combinations of tramadol and paracetamol are available in the Sarajevo canton, Croatia and Slovenia, and morphine in RS, Montenegro, Croatia, Slovenia, Macedonia, and Serbia. Apart from Croatia, morphine cannot be prescribed without consultant’s recommendation. Fentanyl patches are available in Croatia and Slovenia, and spray in Croatia with additional charge (over 30€). Other opioids (oxycodon, pentazocin, buprenofin patches, tapentadol, and combinations) are available only in Slovenia and Croatia.

## Discussion

For years, it has been well known that prevention of illness is the most effective way of health protection. This is especially true for NCDs because prevention does not only lead to health protection but also to reduction of expenses of treatment of illness and its consequences (21–23). Panamerican Health Organisation most efficiently points out to the problem of NCD. The costs of NCDs to the health system, businesses and individuals, are significant and growing. Governments, communities, and private industries are all affected by the high costs of premature death and disability as well as of treatments and caretaking for those living with NCDs. The burden is so great because of the large numbers of people affected, especially those men and women of working-age who are not able to secure productive employment. Without adequate prevention and early detection, these costs only rise, as they require expensive treatments, surgeries, and medications and cut productive lives short. Complications of NCDs incur considerable costs; for example, diabetic nephropathy was estimated as the most costly complication of diabetes in the Americas (22).

Medicines recommended according to the World health’s organization’s Model List of Essential Medicines are included into positive medicine lists of all Western Balkan countries (23). There are a large number of parallels. In most countries, there are unnecessary restrictions regarding prescribing in family practice, what reduce family physicians’ competencies, availability of health care and increase health-care costs.

Most countries and cantons possess their own guidelines for treating hypertension, which were mostly founded on European guidelines (24–28). Quality treatment of hypertension is enabled in all countries and cantons considering the fact that all lists are made of medication mentioned in international guidelines. There is, however, a degree of difficulty, as ARB inhibitors in some countries/cantons cannot be prescribed without consultant’s recommendation, which greatly reduces the level of available health care. Even though we could not find the reasons for this decision made by the fund in every single local guideline, the funds still made such a recommendation. Likewise the recommendation of the Croatian fund that the ARB inhibitor can be introduced after 4 months of coughing is professionally inexplicable. Especially, so as prices of ACE and ARB inhibitors differ by a very small amount. Availability is reduced by increasing waiting lists for examinations and increased costs of transportation to the consultants in case of patients from rural areas. Another problem is that in some countries/cantons combinations of medicine are additionally charged. Considering the poor financial situation for many inhabitants of Western Balkan countries (29), using such medication could greatly burden a patient’s household or reduce compliance as Selmanovic et al. found in their study (30). It would be interesting to explore in what way does a physician make a decision in favor of one brand when there are no restrictions placed by funds (31, 32). All restrictions regarding medication prescribing competency of family physicians involving certain ARB antagonists, diuretics, or beta blockers should be reexamined and adjusted to best evidence-based recommendations (32). Policymakers need to ensure that future reforms will adequately address such financial burden from NCDs and improve access to heahlthcare needed by the population (33).

With the exception of the Herzegovina Neretva canton, medicine for reducing cholesterol and triglycerides are on positive list of all countries/cantons. However, funds’ guidelines are very confusing and are not in accordance with international guidelines. Greater priority to treating hyperlipidemia and improving the accessibility of medicines to treat them should be given. Development and use of evidence-based guidelines for the treatment and efficient procurement and distribution of statins are important mechanisms for providing sustainable access to hyperlipidema (23, 34, 35). Future research could show the effects of the restriction policy regarding statins prescribing on population’s health (36).

Diabetes mellitus type 2 could become the leading public health problem considering the resources necessary for its early diagnosis and treatment (37). All countries and cantons have basic medications for treatment of diabetes, while few also provide newer antidiabetic agents, such as DPP4 inhibitors and SGLT2 inhibitors (which are additionally charged). The basic oral antidiabetic agents are not additionally charged, which helps patient’s budget and increases his adherence. There are important restrictions regarding insulin prescribing-related competencies in family practice, but even in the countries where restrictions are not imposed, family physicians are reluctant to prescribe insulin (33, 38). As emphasized by Kovacevic et al., diabetes morbidity and mortality can be significantly reduced if pharmacotherapy is accessible and affordable (39). It is also necessary to transfer responsibilities for treating type 2 diabetes onto family physicians, with the appropriate education and work quality control.

The greatest restrictions set on family physicians are in the area of treating asthma and COPD. To treat these two diseases in every country and canton, with the exception of Slovenia and Croatia, a recommendation by a clinical consultant is needed. In some Western Balkan countries, inhaled medications are additionally charged (40, 41). We cannot explain why theophylline and aminophyline are left to be prescribed freely by family physicians (considering their narrow therapeutic window), while inhaled corticosteroids are not. Treatment of asthma in family practice is unsatisfactory on a global level, but if these restrictions are kept, family physicians cannot play important role in disease’s control. Data from the PACE program serves as proof that far better results are achieved in treatment of asthma when family physicians take control over patient care (42).

Pain therapy is a basic human right; therefore, it is necessary to remind policymakers that in treating cancer pain there should be no restrictions in prescribing analgesics of all kinds. Likewise, despite limited funds of the health-care system, all countries/cantons should have as great a number of analgesics as possible (43).

Previous studies showed that there is a trend of increasing pharmaceutical expenditure in Balkan countries, what led to the introduction of new policy measures (44). Although analysis of pharmaceutical expenditure represents important perspective of the overall drug utilization, it has only economic side and should be examined within the volume of prescribed drugs (45) as well as through other aspects of pharmaceutical utilization, such as rational prescribing and generic utilization (39, 46).

Jakovljevic and Souliotis found that restrictive policies toward medicines might have negative effects on health care system, creating significant costs to the system or worse health outcomes. The authors also stated that chronic illnesses (e.g., diabetes, COPD, and cancer) serve as the evidence of vulnerabilities, therefore presenting core targets for more responsible, evidence-based national resource allocation strategies (47). Rational use of drugs and rational prescribing are seen as an appropriate way of utilization of limited public resources that might affect pharmaceutical expenditure without compromising the rights of patients to obtain needed medicine (48, 49). Medicines are a dominant part of health system due to necessity to use them in the treatment of disease and high use of available resources in the health care system toward medicines. In addition to problems in jurisdiction conflict and overlaps in countries, significant funds are often spent on medicines that do not have therapeutic value, while there is a deviation in pricing and establishment of control (50). Primary challenge for sustainable funding of prescribed medicines is to manage the difficulties to withstand pressures arising from population aging and high prevalence of NCDs in the Western Balkan countries, what currently increases and will further increase a need for pharmaceuticals or their consumption in the future (51).

There are continuing demands for family physicians to keep the balance between gatekeeper and advocate role, increasingly being confronted with the consequences of allocation policies. Often, it is difficult to integrate gate keeping into heterogeneous family practice and the balance, in that case, cannot be maintained (52). In the countries of Western Balkan, physicians often pay fines if they have spent more money on their patients’ treatment than planned by the contract with Health Insurance Funds, regardless of how many patients with chronic illnesses they saw in their practices or therapeutic indications. As we can see from the results, there are many restrictions on prescribing essential medicines in family practice. In such cases, consultants request to see patients several times per year, with the myriad of laboratory and diagnostic investigations, that family physicians have financial responsibilities for, but, at the same time, are not permitted to participate in decision-making. The question is whether such a policy related to prescribing really permits gate keeping? In addition to medicine reimbursement cost, fee-for-service payments for consultations and additional investigations are very high and unnecessarily burden the health care system. National and international clinical guidelines set up clear, clinical indications for treatment routes of NCDs that family physicians are very well trained in and can practically use to make the best therapeutic decision for their patients. These gaps in global prescribing policies need to be addressed in the future. Knowledge and technologies exist to bring down the burden of NCDs. Paying for NCD prevention and management is an investment (22).

Reimbursement policy based on cost-effectiveness principles and reference pricing by regulatory bodies to manage pharmaceutical costs should be improved in the future (53). Quantity and quality research and comparison of data on pharmaceutical expenditure are needed to explore the impact of different policies in diverse settings, particularly in the countries with limited financial resources (44).

One of the strengths of the current study is that it was performed in the countries with the same legacy toward health-care legislative. This is also the first study exploring prescribing-related competencies of family physicians in Western Balkan countries. Our findings can serve as a basis for further research on prescribing policy and legislation in the region or within other countries. Limitations of the study are those inbuilt with qualitative studies (54). The documents included into analysis are created independent of research question.

## Conclusion

Western Balkan countries are capable of providing essential medicines for the treatment of NCDs, with full or partial reimbursement. There are some exceptions, related to statins, new generation of oral antidiabetic agents and few antihypertensive combinations. Opioid formulations for cancer pain treatment, in the form of codeine, morphine or fentanyl are not available in all countries. Prescribing-related competences of family physicians are limited. However, this practice is not compliant to the practices of family medicine, its principles and primary care structures, and may potentially result in increased health-care financial ramifications to both the system and patients due to frequent referrals to the specialists. Future research in these areas is sorely needed as well as strengthening of family medicine in the region.

## Author Contributions

All the authors have provided substantial contributions to the development of the manuscript. TP-P, MR, and SK contributed to the overall conception and design. TP-P and MR gathered the data. TP-P and MR analyzed the data. All the authors contributed to the interpretation of the data and the drafting of the manuscript. All the authors have given final approval for the paper to be published in Frontiers and agree to be accountable for the content presented therein.

## Conflict of Interest Statement

The authors declare that the research was conducted in the absence of any commercial or financial relationships that could be construed as a potential conflict of interest.
